# Deep Learning-Based Structure-Activity Relationship Modeling for Multi-Category Toxicity Classification: A Case Study of 10K Tox21 Chemicals With High-Throughput Cell-Based Androgen Receptor Bioassay Data

**DOI:** 10.3389/fphys.2019.01044

**Published:** 2019-08-13

**Authors:** Gabriel Idakwo, Sundar Thangapandian, Joseph Luttrell, Zhaoxian Zhou, Chaoyang Zhang, Ping Gong

**Affiliations:** ^1^School of Computing Sciences and Computer Engineering, The University of Southern Mississippi, Hattiesburg, MS, United States; ^2^Environmental Laboratory, U.S. Army Engineer Research and Development Center, Vicksburg, MS, United States

**Keywords:** deep neural networks, deep learning, random forest, androgen receptor, structure-activity relationship, multi-class classification, agonist, antagonist

## Abstract

Deep learning (DL) has attracted the attention of computational toxicologists as it offers a potentially greater power for *in silico* predictive toxicology than existing shallow learning algorithms. However, contradicting reports have been documented. To further explore the advantages of DL over shallow learning, we conducted this case study using two cell-based androgen receptor (AR) activity datasets with 10K chemicals generated from the Tox21 program. A nested double-loop cross-validation approach was adopted along with a stratified sampling strategy for partitioning chemicals of multiple AR activity classes (i.e., agonist, antagonist, inactive, and inconclusive) at the same distribution rates amongst the training, validation and test subsets. Deep neural networks (DNN) and random forest (RF), representing deep and shallow learning algorithms, respectively, were chosen to carry out structure-activity relationship-based chemical toxicity prediction. Results suggest that DNN significantly outperformed RF (*p* < 0.001, ANOVA) by 22–27% for four metrics (precision, recall, F-measure, and AUPRC) and by 11% for another (AUROC). Further in-depth analyses of chemical scaffolding shed insights on structural alerts for AR agonists/antagonists and inactive/inconclusive compounds, which may aid in future drug discovery and improvement of toxicity prediction modeling.

## Introduction

Toxicity caused by chemical exposure can be manifested sequentially at ascending organismal levels, which often begins as a molecular initiating event and escalates into adverse effects measured as toxicological endpoints for the cell, tissue, organ, organism, or population (Ankley et al., 2010; Organization for Economic Co-operation and Development [OECD], 2013; Allen et al., 2014). There exist three categories of chemical toxicity testing strategies: *in vivo, in vitro*, and *in silico*. Due to the prohibitively high costs and ethical concerns over animal welfare associated with *in vitro* and *in vivo* assays, there has been an increasing demand for reduced animal use as well as a shift in toxicity testing paradigms from *in vivo*/*vitro* to *in silico* (National Research Council, 2007). This demand has also been driven by the 3Rs (Replacement, Reduction, Refinement) movement (Stokes, 2015) and by government policies, regulations and legislation [e.g., REACH by the European Union (2006)]. Despite significant advances made in the past decades, *in silico* prediction of chemical toxicity without performing any biochemical (ligand binding) or *in vitro*/*vivo* assays remains an unresolved challenge (Li et al., 2018). Among all *in silico* approaches, structure-activity relationship (SAR)-based modeling has become the predominant one, and it is capable of both qualitative classification and quantitative prediction.

Once the toxicity endpoint or biological activity for prediction is set, the performance of SAR-based predictive modeling is largely determined by the choice of molecular descriptors relevant to toxicity (Shao et al., 2013) and of the prediction modeling algorithms (Plewczynski et al., 2006). The latter varies from linear methods, such as multiple linear regression (MLR), partial least squares (PLS), and linear discriminant analysis (LDA) to non-linear methods, such as *k*-nearest neighbors (KNN), artificial neural networks (ANN), decision trees, and support vector machines (SVM) (Dudek et al., 2006). Recently, deep learning (DL), with the Rectified Linear Unit (ReLU) activation function and such architectures as recurrent neural networks (RNN) and convolutional neural networks (CNN), has emerged as a promising tool for *in silico* toxicity or bioactivity prediction modeling (Hughes et al., 2015, 2016; Xu et al., 2015; Gao et al., 2017; Hughes and Swamidass, 2017; Wu and Wang, 2018). DL, also called deep structured learning or hierarchical learning, allows computational models that are composed of multiple processing layers to be fed with raw data and automatically learn multiple levels of abstract representations of data for performing detection and classification (LeCun et al., 2015). The success of DL has been well documented in such diverse fields as image and speech recognition (Shen et al., 2017; Cummins et al., 2018), visual art (Huang et al., 2016c), natural language processing (Névéol et al., 2018), drug discovery (Dana et al., 2018), bioinformatics (Min et al., 2016), computational biology (Angermueller et al., 2016), and the game of GO (AlphaGo) (Silver et al., 2016).

One of the earliest case studies of applying DL in SAR-based toxicity prediction was reported by Mayr et al. (2016) who developed the DeepTox pipeline. The authors trained deep neural networks (DNNs) using the Tox21 Data Challenge dataset (i.e., training data) that consisted of approximately 12,000 compounds and 12 *in vitro* bioassays (Huang et al., 2016a; Huang and Xia, 2017), and then they predicted the toxicity of approximately 650 chemicals (test data). Although the multi-task DNN exceled in terms of the average AUC (Area Under the Curve of receiver operating characteristics) of the overall 12 bioassays, the nuclear receptor (NR) signaling panel (7 assays), and the stress response (SR) panel (5 assays), it did not perform as well for 5 out of the 12 bioassays as conventional shallow learning techniques did (e.g., SVM, random forest (RF), and elastic net) (Mayr et al., 2016). These results are consistent with the performance of DeepTox in the Tox21 Data Challenge competition where the DeepTox pipeline ranked behind several shallow learning techniques for half of the 12 bioassays even though it won 9 sub-challenges, including those for the other 6 bioassays, the NR and the SR panels, and for the 12 bioassays overall (Mayr et al., 2016; Huang et al., 2016a).

In the past 3 years, more than a dozen papers have been published with conflicting conclusions on comparative performance between DL and shallow learning. For instance, the deepAOT (DL-based acute oral toxicity) models constructed using a molecular graph encoding convolutional neural network (MGE-CNN) architecture outperformed previously reported shallow learning models in both quantitative toxicity prediction and toxicant category classification (Xu et al., 2017). By pairing element specific topological descriptors (ESTDs) with multitask DNN, TopTox (topology-based multitask DNNs) was demonstrated to be more accurate than RF and gradient boosting decision tree (GBDT) using four benchmark ecotoxicity datasets (Wu and Wei, 2018). On the contrary, SVM outperformed DNN in predictive classification of chemical-induced hepatocellular hypertrophy (Ambe et al., 2018), and multiple layer perceptron (MLP) exceeded the performance of 2D ConvNet (2D Convolutional neural network) in the aforementioned 12 Tox21 bioassays (Fernandez et al., 2018). Meanwhile, Liu et al. (2018) found that the overall performance of DNN models was similar to that of RF and variable nearest neighbor methods. They also concluded that neither a larger number of hidden neurons nor a larger number of hidden layers necessarily leads to better neural networks for regression problems. This contradicted previous observations that deeper and wider networks generally performed better than shallower and narrower ones (Koutsoukas et al., 2017; Lenselink et al., 2017). Recently, Mayr et al. (2018) conducted a large-scale comparison of drug target prediction between DL (Feed-forward neural networks or FNN, CNN, and RNN) and shallow learning (RF, SVM, KNN, naïve Bayes (NB), and similarity ensemble approach) methods using a large benchmark dataset (456,331 compounds and more than 1000 assays) from the ChEMBL database. Although FNN was statistically identified as the frontrunner across a wide variety of assay targets, the authors observed that RF and SVM had higher average AUC scores than CNN and RNN.

As a new domain with less than 5 years of application history, we have yet to see overwhelmingly significant and convincingly consistent improvements in both quantitative prediction and qualitative classification of chemical toxicity using DL. Evidence has indicated that DL sometimes does enhance prediction accuracies over shallow learning. However, obtaining such results appears to occur on a case-by-case basis, and the opposite outcomes have also been reported. More studies are warranted to look into many confounding factors such as descriptors, assay targets, chemical space, hyper-parameters, and DL architectures, all of which may impact the performance of DL in QSAR-based chemical toxicity prediction.

Motivated by the aforementioned controversy, we conducted the present study to further investigate if DL algorithms could be optimized to offer a significant improvement over representative shallow learning algorithms for a suite of performance metrics. In the following, we first describe two Tox21 quantitative high throughput screening (qHTS) assay datasets with more than 10,000 compounds. These cell-based qHTS assays were conducted to identify small molecule agonists and antagonists of the androgen receptor (AR) signaling pathway (Huang et al., 2016b). Then, such structural features as 1D–3D molecular descriptors and fingerprints were computed for each chemical. Two algorithms, i.e., DNN (representing DL) and RF (representing shallow learning), were employed to build SAR-based classification models so as to compare the accuracy of these methods for predicting chemical class labels (i.e., agonist, antagonist, inactive, and inconclusive). Our results suggest that DNN outperformed RF not only significantly by statistical analysis, but by a large margin of more than 20% in four of the five performance metrics. Further in-depth analyses of chemical scaffolding shed insights on the structural alerts for the four classes of chemicals in AR activity, which may aid in future drug discovery and improvement of toxicity prediction modeling.

## Materials and Methods

### Bioassay Dataset Curation and Preprocessing

Toxicology in the 21st century (Tox21) is a collaborative initiative launched by the consortium of the NIH, EPA and FDA aiming to develop better toxicity assessment methods^1^. The Tox21 program has tested over 10,000 chemicals against a panel of NR and SR signaling pathways (Attene-Ramos et al., 2013; Huang et al., 2016b). AR, a nuclear hormone receptor, plays a critical role in AR-dependent prostate cancer and other androgen related diseases (Tan et al., 2015). Two *in vitro* assays were carried out in both agonist mode and antagonist mode to assess the agonistic and antagonistic properties of Tox21 chemicals, respectively. The first assay (BLA assay) used the AR-UAS-bla-GripTite^TM^ cell line that contained the ligand-binding domain (LBD) of the rat AR and stably expressed a beta-lactamase reporter gene under the transcriptional control of an upstream activator sequence (UAS). The second assay (MDA assay) used a human breast carcinoma cell line (MDA-kb2 AR-luc) stably transfected with a luciferase reporter gene. A total of 10,496 chemicals were tested, and their assay outcomes were downloaded from the Tox21 Data Challenge website^2^. The downloaded datasets (2 assay modes × 2 assays) were merged using PubChem Substance IDs (SID) because SID was unique for each entry in the datasets. Of the 10,496 compounds, 149 compounds were mixtures of chemicals such as oils and solvents and another 96 compounds contained atoms for which reliable force field parameters were unavailable to perform molecular docking with (see section “Chemical Structure Preparation” below). Thus, these 245 compounds were removed. There was redundancy in the remaining compounds because, on some occasions, multiple SIDs were found corresponding to the same PubChem Compound ID (CID). Hence, CIDs were used to identify and remove redundant chemicals, resulting in 7665 unique chemicals (see Supplementary Figure S1).

For each SID entry, there were up to four records of qualitative assay outcomes that resulted from two assays (BLA and MDA) in two assay modes (agonist and antagonist). There were three possible assay outcomes, i.e., active agonist, active antagonist, or inactive. We assigned one of four class labels, namely “agonist,” “antagonist,” “inactive,” or “inconclusive,” to each chemical by adopting the following rules: a chemical was labeled (i) “agonist” only if both assays in the agonist mode determined it to be an active agonist, (ii) “antagonist” only if both assays in the antagonist mode determined it to be an active antagonist, (iii) “inactive” if all assay outcomes for this chemical were negative, or (iv) “inconclusive” if any other combination was true. In the case of chemical entry redundancy, i.e., multiple SIDs corresponding to the same CID, a consensus was reached on the class label by selecting the most frequently occurring response (i.e., the assay outcome with the highest incidence of occurrence), or the chemical was removed if the assay outcomes were evenly split among multiple categories. Finally, 7665 unique chemicals with unambiguous consensus assay outcomes were obtained and used in the downstream steps (see Supplementary Figure S1).

### Chemical Dataset Curation and Preprocessing

#### Chemical Structure Preparation

The SMILES of the 7665 unique chemicals were downloaded from PubChem via its PUG REST interface^3^ (Kim et al., 2018) using a custom R script. The Open Babel program (O’Boyle et al., 2011) was used to perform the following steps to clean and optimize the downloaded chemical structures (also see Supplementary Figure S1). Salts and other small fragments were removed and only the largest fragment of each entry was retained. SMILES were converted to 2D structures and hydrogens were added when necessary. Then, 3D conformations were generated and partial charges were assigned using the *Electronegativity Equalization Method* followed by energy minimization using the *steepest descent* algorithm (Bultinck et al., 2002; Geidl et al., 2015). Finally, molecular docking was performed to generate biologically relevant 3D ligand conformations within the binding site of the AR because the bound ligand conformation was typically different from the conformations obtained in its unbound state (Tirado-Rives and Jorgensen, 2006; Thangapandian et al., 2010). Molecular docking was performed using the AutoDock Vina program (Trott and Olson, 2010) and the X-ray crystal structure of AR-testosterone complex (PDB ID. 2AM9) (de Jésus-Tran Karine et al., 2006). A cubic box of 16 Å × 16 Å × 16 Å centered at the binding site was used to dock the chemicals in the data set. The docking-generated ligand conformations were used for 3D descriptor calculations (see section “Feature Generation and Dimensionality Reduction” below).

#### Feature Generation and Dimensionality Reduction

A total of 17,967 molecular descriptors and fingerprints (termed features) were generated using PaDEL (Yap, 2011), including 1444 1D or 2D descriptors, 431 3D descriptors, and 16,092 unique fingerprints belonging to 12 different pattern types. The 3D descriptors were calculated using the binding conformations obtained above from molecular docking. In case PaDel failed to compute certain features for certain compounds, the mean-imputation method as implemented in Scikit-Learn (Pedregosa et al., 2011) was employed to replace those missing values. A variance thresholding method was used to reduce feature dimensionality. Any feature vector with at least 85% of its entries being identical was removed, resulting in a final set of 2544 features.

#### Feature Standardization

For many algorithms, it is necessary to rescale the features to keep certain features from getting more influence than they should. This particularly holds true for neural networks where certain weights may update faster than others, thus making optimization methods converge less quickly (LeCun et al., 2015). Also, the generated features were of varying scales and distributions, and they were also comprised of count and binary features. To resolve this, the features in the final set were standardized (rescaled) individually such that they assumed a standard normal distribution with a mean of zero and unit standard deviation. Using the StandardScaler function in Scikit–Learn (Pedregosa et al., 2011), the training dataset was rescaled by subtracting the mean and dividing the resulting difference by the standard deviation. The mean and standard deviation used in the training dataset were used to transform the test dataset.

#### Chemical Space Visualization

The chemical space of the 7665 unique Tox21 chemicals was visualized in two-dimensional vectors. The space of the final set of 2544 features was further reduced to two abstract features using an autoencoder (Baldi, 2012; Chandra and Sharma, 2015). By trying to reconstruct the input at the output layer, the autoencoder was forced to learn the underlying feature space in a lower dimension. The innermost layer of the autoencoder, an embedding of the input, was set to two units. The encoder component of the autoencoder had 2544 units in the input layer corresponding to the number of features in the input data and {1024, 512, 128, 32, 2} features in the hidden layers. The decoder component of the autoencoder was ordered as the reverse of the encoder. For activation functions, ReLU was used in the hidden layers while sigmoid functions were used in the output layer. The Adam optimizer was used to minimize the mean squared error. The autoencoder model was trained using the Keras (Chollet, 2015) Python library with a Tensorflow backend.

### Machine Learning Methods

#### Machine Learning-Based SAR Modeling Approach

**FIGURE 1:**
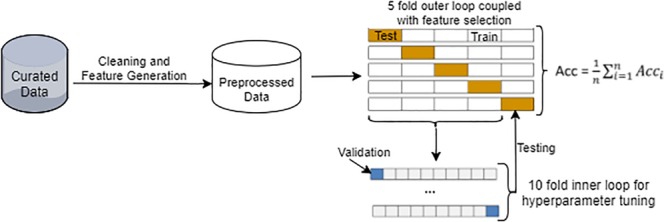
Overview of the machine learning-based SAR approach with a nested double-loop cross-validation strategy for model construction, validation, and evaluation. Details of data preprocessing are provided in Supplementary Figure S1.

The overall workflow of our machine learning-based SAR modeling approach is illustrated in Figure 1. It began with data curation, followed by preprocessing of chemical structure and *in vitro* assay data. We employed a nested double-loop cross-validation strategy to ensure robust model development and to alleviate the impact of selection bias and overfitting (Cawley and Talbot, 2010). Similar to most other typical SAR datasets, the 7665 unique chemicals displayed an imbalanced distribution across the four assay outcome classes, i.e., agonist, antagonist, inactive, and inconclusive. As a result of the imbalance, a stratified sampling strategy was adopted to ensure that the partitioning of chemicals across all classes remained the same between the cross-validation folds and between the training and test datasets.

The 7665 chemicals were split randomly using the stratified strategy into five subsets. For each run of the outer loop, one subset (20%) was withheld as the test set while the remaining four subsets (80%) were used as the training set. Each of the five runs in the outer loop used a different subset. In the inner loop, the training set was further randomly split into 10-folds using the stratified strategy. Ninefolds were used for model (classifier) training or hyper-parameter tuning, while the remaining onefold was used for validation. Thus, a 10-fold cross-validation was implemented in the inner loop for classifier training, whereas a fivefold cross-validation was executed in the outer loop for model testing and evaluation. The overall performance was assessed using the average metrics values of all five runs in the outer loop (see section “Chemical Scaffolding and Similarity Analysis” for metrics definition).

#### Shallow and Deep Learning Algorithms

Six commonly used and popular machine learning algorithms were compared in a preliminary study. They included KNN, RF, classification and regression trees (CART), NB, SVM, and DNN, all of which ran under their respective default settings as implemented in Scikit-Learn (Pedregosa et al., 2011). Their performance without optimization was determined by following the workflow presented in Figure 1. Based on their performance metrics as shown in Supplementary Figure S2, we selected the top two algorithms, DNN and RF, for further optimization and chemical toxicity classification in this study.

##### Random forest and optimization

Random forests are a collection of decision trees whose predictions are averaged to obtain an ensemble performance. Randomness is achieved by allowing each tree in the forest to use bootstrap samples of the training data and random molecular features selection for prediction. Decision Trees are drawn upside down and begin with a trunk that splits into multiple branches before eventually arriving at the leaves. The leaf nodes represent the endpoint to be predicted, while all other nodes are assigned a molecular feature. To construct a robust decision tree, the features (nodes) that most clearly differentiate the endpoints (leaf nodes) are chosen. *Gridsearch* with 10-fold cross validation was employed in optimizing the RF models. The distribution of parameters optimized for the RF model is provided in Supplementary Table S1.

##### Deep learning and optimization

###### Deep learning architecture

**FIGURE 2:**
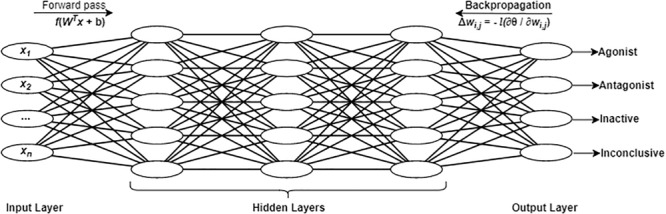
A fully connected deep neural network with an input layer, three hidden layers, and an output layer. As an example, four units are shown in the output layer corresponding to the four chemical activity classes in the present study.

We briefly describe this algorithm and the hyper-parameters of DNNs in order to facilitate our discussion of the optimization and performance analysis process. A DNN is an artificial neural network with one input layer, multiple hidden layers and one output layer, as shown in Figure 2. The number of hidden layers is defined as *k*. Each layer consists of a number of units (or neurons), denoted by *n*. The number of units at the input layer corresponds to the number of features in the input data (*x*). The number of units in the output layers is equal to the number of classes to be predicted. In this study, there were four units in the output layer that corresponded to four classes: (i) agonist, (ii) antagonist, (iii) inactive, and (iv) inconclusive. The number of units in each hidden layer usually depends on specific details of various classification problems and datasets. Typically, it is determined by multiple trials of different network topologies. For a fully connected network as used for this study, each pair of units *i* and *j* in two consecutive layers are connected by a link with a weight *W*_*i,j*_. There is an input and an output for each unit. In the input layer, the output is the same as the input for each unit. For each unit in the hidden layer, the input is comprised of the weighted sum of the units in the previous layers and the bias of the current unit. The output of each hidden layer unit is obtained by applying an activation function to its input. The ReLU activation function is applied to all units in all the hidden layers and computes the function *f*(*x*) = max(0,*x*). This allows for easy gradient computation, which in turn results in faster training for large networks. By feeding the training data in batches to the input layer (with a specified batch size), the DNN with a given network topology and weights can compute the predictions in the output layer. During the training process, a dropout regularization technique is used to ignore some randomly selected neurons in order to prevent the neural networks from overfitting. Dropout rate is a parameter that needs to be tuned in DL. The softmax function is applied to the output layer to obtain a categorical probability distribution with values between 0 and 1, indicating the likelihood that any of the four classes are true. The highest probability determines the class label of each sample.

###### Learning process

Training a neural network with a given architecture is a process performed to find a combination of weights of units so as to minimize the error between the predictions in the output layer and the known truth. In our study, categorical cross entropy θ is used as the loss function to compute the error. We can minimize the objective function θ by iteratively applying optimization methods such as mini-batch gradient descent, Adam, RMSprop, and Adagrad. Backpropagation is used in gradient descent methods to update the weights of units by computing the gradient ∇θ of the loss function with respect to weight *W*_*i,j*_.

The weights are updated in the opposite direction of ∇θ. The update of the weight *w*_*i,j*_ is defined as $\Delta \,w_{i,j}=-l\,\frac{\partial \theta }{\partial w_{i,j}}$

where *l* refers to the learning rate that determines the size of the steps taken at each iteration to reach the minimum of the objective function. The weights are updated iteratively, and the learning process repeats until the neural networks are trained adequately. This means that the loss function decreases to a certain threshold.

###### Hyper-parameter optimization

The hyper-parameters in DL need to be tuned to get the best model suited for the dataset. These hyper-parameters include the number of hidden layers, the number of units in the input layer, the number of units in the hidden layers, the number of units in the output layer (e.g., set to 4 in this study because of the four categories of the chemical activity classification), batch size, dropout rate, learning rate and optimizer.

Bayesian hyper-parameter optimization has been shown to perform faster and more accurately than grid and random parameter search, respectively (Snoek et al., 2012). The rationale for Bayesian optimization is to liken the optimization of hyper-parameters to a function minimization challenge. In Bayesian hyper-parameter optimization, a probability model of the objective function is constructed, which is often referred to as a surrogate function and denoted as *p*(score | parameters). Instead of randomly selecting parameters or going through a grid in a blind manner, the results of the surrogate function are used to select the next parameters to try on the objective function, thus minimizing the number of calls to the objective function. The hyper-parameters with the best score or least validation set error computed by the objective function are considered the optimal. In this study, the search for optimal hyper-parameters was conducted using Bayesian optimization as implemented in Hyperas, a tool that combines the Keras DL library (Chollet, 2015) with Hyperopt’s Sequential Model-Based Optimization (SMBO) methods using the Tree-structured Parzen Estimator (TPE) algorithm (Bergstra et al., 2011). The search space included hidden layers {2,3,4}, Neurons {32,64,128,256,512,1024}, optimization methods {mini-batch gradient descent, Adam, RMSprop, Adagrad}, batch size {8,16,32,64,128}, and learning rate {random uniform distribution between 0 and 1}.

#### Model Evaluation Metrics

Five metrics were computed for model performance evaluation. They included precision, recall, F1-score (also called F-measure), the area under the receiver operating characteristic curve (AUROC), and the area under the precision-recall curve (AURPC). Macro-averages of the performance metrics were calculated and used for evaluation throughout this study because of the imbalanced nature of the data and the multi-category classification task. Macro-averaging independently computes the average for every class prior to averaging. By giving the same weight to all classes, it can show how effective a model is on the minority classes, e.g., AR agonists and AR antagonists that are of greater importance in this study. Micro-averaging was not considered as it gives equal weight to every sample; hence, the majority classes contribute more to the average metric than the minority classes. The following formulas describe computing the macro-averages of precision, recall and F-measure.

$${\text{Precision}}_{\text{macro }}=\frac{\sum_{i=1}^{m}\frac{t\,p_{i}}{t\,p_{i}+f\,p_{i}}}{m}$$

$${\text{Recall}}_{\text{macro }}=\frac{\sum_{i=1}^{m}\frac{t\,p_{i}}{t\,p_{i}+f\,n_{i}}}{m}$$

$$F-{\mathrm{measure}}_{\text{macro }}=\frac{\sum_{i=1}^{m}\left(\frac{2\times {\text{Precision }}_{i}\times {\text{Recall }}_{i}}{{\text{Precision }}_{i}+{\text{Recall }}_{i}}\right)}{m}$$

where *m* = number of classes, *tp* = true positive, *fp* = false positive, *fn* = false negative.

The AUROC and the AUPRC were determined in Scikit–Learn (Pedregosa et al., 2011) by computing the area under the plot of true positive rate vs. false positive rate and that of precision vs. recall, respectively. The macro-averages of AUROC and AUPRC were calculated in a similar fashion to those of precision and recall above.

#### Implementation Environment

The machine learning models were developed in Python 3.5.4 using Jupyter Notebook within the Anaconda 4.3.27 (64-bit) environment. Other important libraries include Scikit-Learn 0.19.0, Keras 2.1.4, Tensorflow 1.9, and Hyperas 0.4. All models were trained on a server (Intel Xeon E5-1650) running Ubuntu 16.04.5 LTS with six cores, 32GB memory and four Nvidia Titan Xp GPUs.

### Chemical Scaffolding and Similarity Analysis

Chemical scaffolding and similarity analysis were performed on one of the five chemical subsets used as the external test set in the first run (i.e., Fold 1 as seen in Figure 1 and Supplementary Table S2). The R packages *Rcdk* and *Rcpi* were used for calculating chemical scaffolds and similarity analysis, respectively. The true labels (not predicted labels) of chemicals were used for both analyses.

In chemical scaffolding, the structural information of a chemical can be organized into rings and frameworks (Bemis and Murcko, 1996). Any cycles that share an edge are defined as rings, whereas any unions of rings via linkers are defined as frameworks. For instance, benzene, naphthalene, and anthracene are single ring systems, whereas diphenylmethane is a framework. Using Murcko chemical scaffolding, a list of rings and frameworks present in the test chemicals was generated.

The Tanimoto coefficient or scores (Bajusz et al., 2015) are a widely accepted metric for evaluating similarity between two chemicals. We calculated the Tanimoto scores, using the PubChem fingerprints as the input, for every interclass pairing (e.g., an agonist vs. an antagonist, an agonist vs. an inactive, an antagonist vs. an inconclusive) in order to compare interclass similarity. The score of 0.5 was selected as the cutoff threshold, i.e., any pairs of chemicals with a score ≥ 0.5 were considered similar to each other.

## Results and Discussion

### Data Distribution and Evaluation Metrics

**FIGURE 3:**
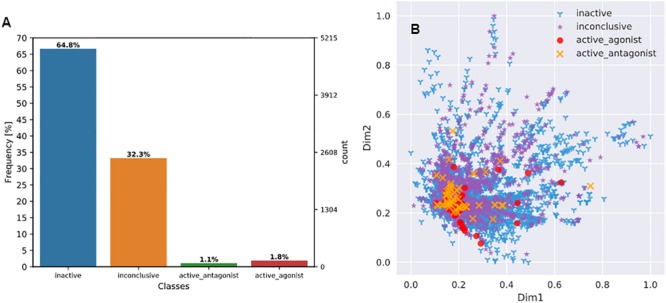
Visualization of chemical distribution over activity classes and chemical space. **(A)** Distribution of Tox21 compounds across four AR activity classes; **(B)** Distribution of all four AR activity classes of compounds over the compressed 2-dimension chemical space. Feature dimensionality was reduced from 2544 to 2 using an autoencoder (see section “Chemical Space Visualization” for more details).

As shown in Figure 3A, the 7665 unique compounds were unevenly distributed across four AR activity classes with the two active classes (222 compounds) being the minority (2.9%) and the inactive (2476) or inconclusive (4967) classes being the majority (97.1%).

An autoencoder was used to reduce chemical feature dimensionality. As a result, the chemical space distribution of the final set of 7665 compounds can be visualized in a 2D plot (Figure 3B). The plot shows that no class forms a distinct cluster, the two inactive classes are more widely dispersed than the two active classes, and that all the active compounds reside within the space of inactive or inconclusive ones. These observations suggest that it was a challenging task to separate the four classes based on the structural features of the compounds.

Owing to the skewed class distribution, one of our main objectives was to develop a classification model with high performance for the minority classes because the two less populated active classes were of higher toxicological importance. Meanwhile, the model should not sacrifice the accuracy of the more abundant inactive and inconclusive classes, which would compromise the overall prediction performance for the entire dataset. Therefore, we chose to use macro-averages over micro-averages (see section “Model Evaluation Metrics” above) and selected evaluation metrics that are sensitive to class imbalance or favorable to minority classes such as F-measure and AUPRC (Jeni et al., 2013). F-measure is considered a better metric than precision (P) and recall (R) because it is a harmonic mean of P and R and also a tradeoff between P and R (Powers, 2011). Although AUROC and AUPRC both provide model-wide evaluation, a classifier that optimizes the area under ROC is not guaranteed to result in an optimal AUPRC (Davis and Goadrich, 2006). When the positives are the minority and more important than the negatives, AUROC is an overly optimistic measure of model performance, whereas AUPRC provides a more informative and accurate depiction of model prediction performance as it evaluates the fraction of true positives among positive predictions (Saito et al., 2015).

### Performance Comparison Between DNN and RF

Only F-measure was determined in the preliminary performance study of six machine learning algorithms without parameter optimization, and RF showed the highest F-measure with a low variance (Supplementary Figure S2). Therefore, RF was selected to represent shallow learning algorithms for further optimization as well as to compare with DNN.

Following the workflow depicted in Figure 1, we optimized the hyper-parameters, built multi-class prediction models, and assessed the model performance. Details of the hyper-parameter optimization approach for RF and DNN are described earlier in section “Shallow and Deep Learning Algoritijhms.” The optimized parameters for RF are provided in Supplementary Table S1. For DNN, we found that (a) the architecture of the best performing classifier had three hidden layers with (1024,1024,512) units; (b) regularization was achieved using dropout rates of (0.25, 0.341, and 0.5) applied on these three hidden layers, respectively; and (c) Mini-Batch Gradient Descent with a batch size of 16 allowed for frequent updates in the weights of the network and a more robust convergence.

**FIGURE 4:**
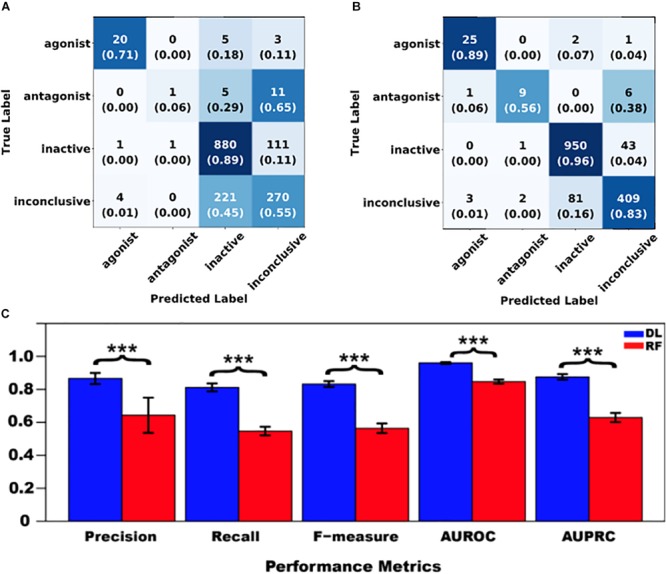
Performance comparison between shallow learning algorithms represented by random forest (RF) and deep learning (DL) algorithms represented by deep neural networks (DNN). **(A)** RF confusion matrix; **(B)** DNN confusion matrix; and **(C)** Metrics comparison [mean ± standard deviation, *n* = 5; Here “^∗∗∗^” stands for statistical significance at *p* < 0.001 (ANOVA, Tukey *post hoc* test)]. In confusion matrices, average numbers of predicted compounds and average recall scores (in parenthesis) for all four classes are shown, and all the cells are colored with a blue gradient (i.e., the darkness increases with the values).

Then, DNN and RF models were separately trained using the same preprocessed data. Figures 4A,B present the confusion matrices and the average recall scores for all four classes calculated from the external fivefold cross-validation (see Supplementary Tables S2–S6 for detailed reports for folds 1–5, respectively). Figure 4C provides the average performance metrics for DNN and RF side-by-side (see Supplementary Tables S7, S8 for the raw metrics data for all fivefolds). These results clearly indicate that DNN consistently outperformed RF in both of the following measures: (1) the average number of correctly classified compounds (recall) for all four classes (Figures 4A,B), and (2) the macro-averages of all five performance metrics across all four classes (Figure 4C).

Specifically, DNN correctly predicted 50% more antagonists and 28% more inconclusive compounds than RF did, whereas the other two classes were not improved as much (i.e., 18% for agonists and 7% for inactive compounds) (Figures 4A,B). Furthermore, the performance enhancement was statistically significant (*p* < 0.001, ANOVA) for each metric (Figure 4C), regardless of whether the metric is insensitive (AUROC) or sensitive (the other four metrics) to imbalanced class distribution (Jeni et al., 2013). It is worth noting that the four imbalance-sensitive metrics were improved by 22–27%, while AUROC was boosted by only 11%. The coefficient of variation (CV = standard deviation/mean) for each metric was less than 5% except for the precision of RF (17%), suggesting that both DNN and RF models had stable performance (Supplementary Tables S7, S8). However, the performance of DNN models was more stable than that of RF (as reflected by much smaller CVs shown in Supplementary Tables S7, S8 and lower error bars seen in Figure 4C).

However, performance did not differ between RF and DNN prior to hyper-parameter optimization in terms of F-measure: 0.548 ± 0.038 for RF vs. 0.536 ± 0.052 for DNN (*p* = 0.654, paired *t*-test; see Supplementary Figure S2). Parameter optimization did not enhance RF performance (F-measure): 0.548 ± 0.038 pre-optimization (Supplementary Figure S2) vs. 0.564 ± 0.029 post-optimization (Figure 4C and Supplementary Table S8) (*p* = 0.579, paired *t*-test). This was due to the fact that the default parameters for RF in Scikit–Learn were not arbitrary (i.e., they are pre-optimized for normal tasks) and were similar or comparable to the selected optimal ones (see Supplementary Table S1). On the contrary, hyper-parameter tuning greatly contributed to the improvement of DNN performance as reflected in the F-measure: 0.536 ± 0.052 pre-optimization (Supplementary Figure S2) vs. 0.832 ± 0.018 post-optimization (Figure 4C and Supplementary Table S7) (*p* < 0.001, paired *t*-test). It has come to our attention that some studies (e.g., Ambe et al., 2018; Fernandez et al., 2018) where suboptimal performance of DL was reported in comparison with shallow learning did not conduct adequate hyper-parameter optimization. These studies along with our own demonstrate the dependence of DL performance on hyper-parameter optimization.

### Chemical Scaffolding Analysis

Using the chemicals in Fold 1 (20% of the entire preprocessed dataset) as an example, we conducted scaffolding analysis. Class-wise Murcko decomposition has revealed that the majority of chemicals contain single-ring systems and no Murcko frameworks (Supplementary Figure S3). Only 2 out of 28 agonists and 3 out of 17 antagonists contain scaffolding systems with more than one ring. These single-ring systems predominantly contain cyclopentanophenanthrene, a fused 4-membered ring system like in testosterone. About 20–30% inactive and inconclusive compounds contain systems with 2–4 rings (Supplementary Figure S3A). Both agonists and antagonists displayed a maximum of only three frameworks, whereas inactive and inconclusive compounds contained as many as 16 frameworks. This meant that the AR active compounds were more compact than the other two classes (Supplementary Figure S3B).

**FIGURE 5:**
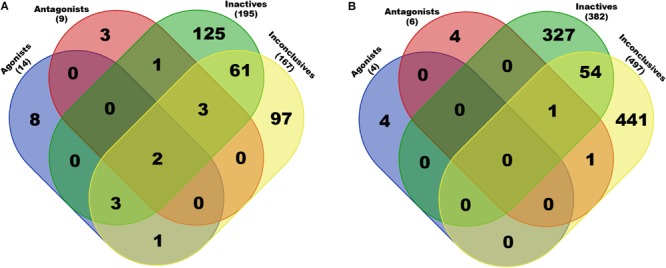
Breakdown of exclusive and shared rings **(A)** and frameworks **(B)** present in each chemical class of AR activity. Only chemicals in the Fold 1 subset (20% of the final set of preprocessed compounds) were used in this analysis. Total numbers of non-redundant scaffolds are given in parentheses (also see Table 1).

**FIGURE 6:**
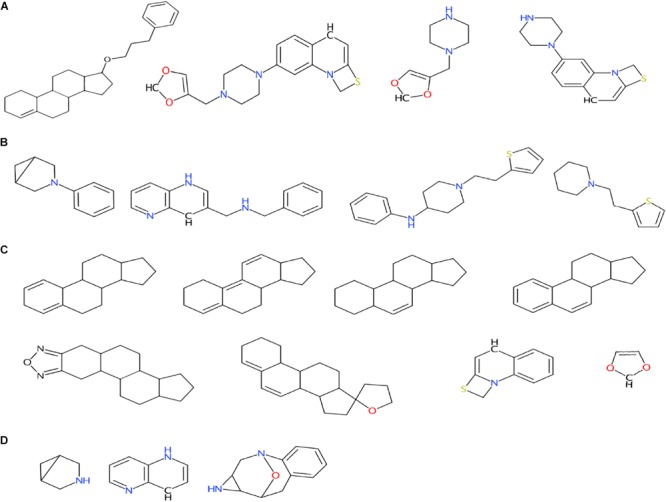
Murcko frameworks exclusively present in agonists **(A)** and antagonists **(B)** as well as Murcko rings exclusively present in agonists **(C)** and antagonists **(D)**. Also see Figure 5 for the numbers of class-specific frameworks and rings for these two classes.

The obtained scaffolds (both rings and frameworks) were compared to explain the differences in prediction accuracy between different classes. The decomposed Murcko rings and frameworks revealed the total and unique chemical backbones present in each class (Table 1) as well as the class-specific backbones and those shared between classes (Figure 5). We identified 8 and 3 class-specific rings for AR agonists and antagonists, respectively (Figurejn 5A), as well as four frameworks unique to these two AR active classes (Figure 5B). Among the 4 agonist-specific frameworks, the 1,3-dioxole (a five-membered heterocycle consisting of two oxygen atoms at the 1 and 3 positions) and thiozetoquinoline (quinoline fused to a four-membered 1,3-thiazetidine) rings are each present in two frameworks, whereas piperazine (a six-membered ring containing two nitrogen atoms at para positions in the ring) is present in three frameworks (Figure 6A). A higher structural diversity is displayed in the antagonist-exclusive frameworks, including *N*-phenyl-azobicyclohexane-, naphthyridine-, piperidine-, and thiophene-containing frameworks, with only the structure of thiazole and piperidine connected by an ethyl linker present in two frameworks (Figure 6B). The 8 agonist- and 3 antagonist-specific rings are shown in Figures 6C,D, respectively. The low scaffold overlapping between agonists and antagonists (2 rings and 0 framework, Figures 5A,B) may explain why these two classes were rarely mistaken for each other during classification (Figures 4A,B). Furthermore, these class-specific scaffolds may serve as potential structural alerts for AR agonists or antagonists and as additional features in future machine learning-based classification or quantitative prediction modeling.

**TABLE 1:** Numbers of total and non-redundant Murcko rings and frameworks present in the Fold 1 subset of Tox21 compounds.

	**Rings**		**Frameworks**	
**Class**	**Total**	**Unique**	**Total**	**Unique**
Agonist	30	14	4	4
Antagonist	20	9	7	6
Inactive	932	195	471	382
Inconclusive	648	167	611	497

**FIGURE 7:**
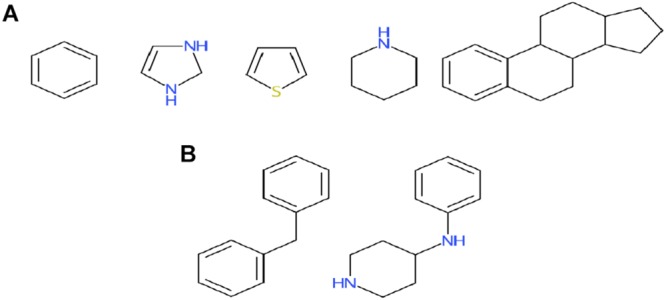
Murcko rings **(A)** and frameworks **(B)** present in both antagonists and inconclusive compounds. Also see Figure 5 for the breakdown of scaffolds among classes.

Among the four classes of chemicals, 65% (Figure 4A) vs. 38% (Figure 4B) of antagonists were misclassified as inconclusive compounds by RF and DNN, respectively; whereas 45% (Figure 4A) vs. 16% (Figure 4B) of inactive compounds were wrongly predicted to be inconclusive compounds by RF and DNN, respectively. These high rates of misclassification may be attributed to the high rates of non-redundant rings (5/9) and frameworks (2/6) present in antagonists that also appear in inconclusive compounds, and of non-redundant scaffolds (69/195 rings and 55/382 frameworks) in inactive compounds overlapping with those in inconclusive compounds (Figure 5). For instance, the overlapping scaffolds between antagonist and inconclusive classes include five rings (benzene, pyrazoline, thiophene, piperidine and reduced cyclopenta[a]phenanthrene) (Figure 7A), and two frameworks (diphenylmethane and 4-phenylamino-piperidine) (Figure 7B). These overlapping scaffolds may confound the learning process in classification modeling, leading to lower prediction accuracies.

### Chemical Similarity Analysis

The Tanimoto scores (TS) determined using PubChem fingerprints have revealed the degree of chemical similarity among the four AR activity classes. For the Fold-1 subset of Tox21 compounds, we determined five types of inter-class, pairwise chemical similarity: agonist-inactive, agonist-inconclusive, antagonist-inactive, antagonist-inconclusive, and agonist-antagonist (Supplementary Figure S4). It was observed that 4.1% (=1133/(28 × 994)) of agonist-inactive pairs and 4.0% (=544/(496 × 28)) of agonist-inconclusive pairs were chemically similar (TS ≥ 0.5), whereas 11.9% (=1788/(17 × 994)) of antagonist-inactive pairs and 10.5% (=875/(17 × 496)) of antagonist-inconclusive pairs were 50% or more similar (Table 2). Similar to scaffolding analysis results, the higher degree of chemical property similarity between antagonists and inconclusive or inactive compounds may have contributed to the high misclassification rates of antagonists (Figures 4A,B). In contrast, agonists, chemically less similar to inactive and inconclusive classes, were predicted with a much higher accuracy than antagonists (Figures 4A,B). The mean Tanimoto scores did not differ significantly among the four types of comparisons, likely due to an equalizing effect caused by high numbers of less similar chemical pairs (Supplementary Figure S4).

**TABLE 2:** Pairwise Tanimoto scores (TS) between active and inactive/inconclusive classes in the Fold-1 subset of Tox21 compounds, consisting of 28 agonists, 17 antagonists, 994 inactive chemicals, and 496 inconclusive chemicals.

	**Inactive (994)**			**Inconclusive (496)**		
	**# Pairs with TS ≥ 0.5**	**Mean TS**	**% in all pairs**	**# Pairs with TS ≥ 0.5**	**Mean TS**	**% in all pairs**
Agonist (28)	1133	0.25 (±0.13)	4.1	544	0.29 (±0.13)	4.0
Antagonist (17)	1788	0.26 (±0.16)	11.9	875	0.31 (±0.17)	10.5

*Shown here are the number of pairs with TS ≥ 0.5 and the percent of these pairs in the total number of possible pairs.*

## Conclusion

Using the multi-class AR dataset from the Tox21 Data Challenge, we conducted a case study to demonstrate that DL (represented by DNNs) was far superior to shallow learning (represented by RFs) for predicting their AR activities. Our results suggest that the performance of DNN was highly dependent on hyper-parameter optimization. Meanwhile, appropriate data preprocessing (e.g., feature generation and standardization), stratified data splitting, a double-loop cross-validation strategy and performance evaluation metrics also played an important role in ensuring high quality data, avoiding over-fitting, and alleviating the impact of skewed class distribution. By performing scaffolding and similarity analyses, we discovered potential causes for antagonists being frequently misclassified as inconclusive or inactive compounds and for inactive compounds being wrongly predicted as inconclusive compounds. The high similarity in chemical properties and structural scaffolding between antagonist and inconclusive compounds and between inactive and inconclusive compounds was identified as a confounding factor that impaired classifier performance. Meanwhile, a number of class-specific scaffolds have been identified as candidate structural alerts for AR agonists and antagonist, which may serve as additional chemical features to improve prediction performance in future studies.

## Author Contributions

PG and CZ conceived and supervised the study. GI and JL conducted the machine learning experiments. ST performed the data preprocessing, chemical scaffolding, and similarity analyses. ZZ and JL carried out the literature survey. All authors contributed to the manuscript writing and revision.

## Conflict of Interest Statement

The authors declare that the research was conducted in the absence of any commercial or financial relationships that could be construed as a potential conflict of interest.

## Footnotes

1 https://ncats.nih.gov/tox21/about/goals

2 https://tripod.nih.gov/tox21

3 https://pubchemdocs.ncbi.nlm.nih.gov/pug-rest

## References

[B1] Allen T. E. H. Goodman J. M. Gutsell S. Russell P. J. (2014). Defining molecular initiating events in the adverse outcome pathway framework for risk assessment. *Chem. Res. Toxicol.* 27 2100–2112. 10.1021/tx500345j 25354311

[B2] Ambe K. Ishihara K. Ochibe T. Ohya K. Tamura S. Inoue K. (2018). In silico prediction of chemical-induced hepatocellular hypertrophy using molecular descriptors. *Toxicol. Sci.* 162 667–675. 10.1093/toxsci/kfx287 29309657

[B3] Angermueller C. Pärnamaa T. Parts L. Stegle O. (2016). Deep learning for computational biology. *Mol. Syst. Biol.* 12:878. 10.15252/msb.20156651 27474269 PMC4965871

[B4] Ankley G. T. Bennett R. S. Erickson R. J. Hoff D. J. Hornung M. W. Johnson R. D. (2010). Adverse outcome pathways: a conceptual framework to support ecotoxicology research and risk assessment. *Environ. Toxicol. Chem.* 29 730–741. 10.1002/etc.34 20821501

[B5] Attene-Ramos M. S. Miller N. Huang R. Michael S. Itkin M. Kavlock R. J. (2013). The Tox21 robotic platform for the assessment of environmental chemicals – from vision to reality. *Drug Discov. Today* 18 716–723. 10.1016/J.DRUDIS.2013.05.015 23732176 PMC3771082

[B6] Bajusz D. Rácz A. Héberger K. (2015). Why is Tanimoto index an appropriate choice for fingerprint-based similarity calculations? *J. Cheminform.* 7:20. 10.1186/s13321-015-0069-3 26052348 PMC4456712

[B7] Baldi P. (2012). “Autoencoders, Unsupervised Learning, and Deep architectures,” in *Proceedings of ICML Workshop on Unsupervised and Transfer Learning* (Washington, DC).

[B8] Bemis G. W. Murcko M. A. (1996). The properties of known drugs. 1. Molecular frameworks. *J. Med. Chem.* 39 2887–2893. 10.1021/jm9602928 8709122

[B9] Bergstra J. Bardenet R. Bengio Y. Kégl B. (2011). “Algorithms for hyper-parameter optimization,” in *Proceeding of the 25th Annual Conference on Advances in Neural Information Processing Systems (NIPS)* (Granada) 2546–2554.

[B10] Bultinck P. Langenaeker W. Lahorte P. De Proft F. Geerlings P. Waroquier M. (2002). The electronegativity equalization method I: parametrization and validation for atomic charge calculations. *J. Phys. Chem. A* 106 7887–7894. 10.1021/jp0205463

[B11] Cawley G. C. Talbot N. L. C. (2010). *On Over-fitting in Model Selection and Subsequent Selection Bias in Performance Evaluation.* Available at: http://jmlr.csail.mit.edu/papers/volume11/cawley10a/cawley10a.pdf (accessed October 21, 2018)

[B12] Chandra B. Sharma R. K. (2015). “Exploring autoencoders for unsupervised feature selection,” in *Proceedings of the 2015 International Joint Conference on Neural Networks (IJCNN)* (Killarney: IEEE) 1–6. 10.1109/IJCNN.2015.7280391

[B13] Chollet F. (2015). *Keras.* Available at: https://github.com/keras-team/keras (accessed October 17, 2018).

[B14] Cummins N. Baird A. Schuller B. W. (2018). Speech analysis for health: current state-of-the-art and the increasing impact of deep learning. *Methods* 151 41–54. 10.1016/j.ymeth.2018.07.007 30099083

[B15] Dana D. Gadhiya S. St. Surin L. Li D. Naaz F. Ali Q. (2018). Deep learning in drug discovery and medicine; scratching the surface. *Molecules* 23:E2384. 10.3390/molecules23092384 30231499 PMC6225282

[B16] Davis J. Goadrich M. (2006). “The relationship between precision-recall and ROC curves,” in *Proceedings of the 23rd International Conference on Machine learning*, (New York, NY: ACM) 233–240.

[B17] de Jésus-Tran Karine P. Pierre-Luc C. Line C. Jonathan B. Fernand L. Rock B. (2006). Comparison of crystal structures of human androgen receptor ligand-binding domain complexed with various agonists reveals molecular determinants responsible for binding affinity. *Protein Sci.* 15 987–999. 10.1110/ps.051905906 16641486 PMC2242507

[B18] Dudek A. Z. Arodz T. Gálvez J. (2006). Computational methods in developing quantitative structure-activity relationships (QSAR): a review. *Comb. Chem. High Throughput Screen.* 9 213–228. 10.2174/138620706776055539 16533155

[B19] European Union (2006). *Regulation (EC) No 1907/2006 - Registration, Evaluation, Authorisation and Restriction of Chemicals (REACH).* Available at: https://osha.europa.eu/en/legislation/directives/regulation-ec-no-1907-2006-of-the-european-parliament-and-of-the-council (accessed June 3, 2018).

[B20] Fernandez M. Ban F. Woo G. Hsing M. Yamazaki T. LeBlanc E. (2018). Toxic colors: the use of deep learning for predicting toxicity of compounds merely from their graphic images. *J. Chem. Inf. Model.* 58 1533–1543. 10.1021/acs.jcim.8b00338 30063345

[B21] Gao M. Igata H. Takeuchi A. Sato K. Ikegaya Y. (2017). Machine learning-based prediction of adverse drug effects: an example of seizure-inducing compounds. *J. Pharmacol. Sci.* 133 70–78. 10.1016/j.jphs.2017.01.003 28215473

[B22] Geidl S. Bouchal T. Raček T. Svobodová Vareková R. Hejret V. Krenek A. (2015). High-quality and universal empirical atomic charges for chemoinformatics applications. *J. Cheminform.* 7:59. 10.1186/s13321-015-0107-1 26633997 PMC4667495

[B23] Huang R. Xia M. (2017). Editorial: Tox21 challenge to build predictive models of nuclear receptor and stress response pathways as mediated by exposure to environmental toxicants and drugs. *Front. Environ. Sci.* 5:3 10.3389/fenvs.2017.00003

[B24] Huang R. Xia M. Nguyen D.-T. Zhao T. Sakamuru S. Zhao J. (2016a). Tox21Challenge to build predictive models of nuclear receptor and stress response pathways as mediated by exposure to environmental chemicals and drugs. *Front. Environ. Sci.* 3:85 10.3389/fenvs.2015.00085

[B25] Huang R. Xia M. Sakamuru S. Zhao J. Shahane S. A. Attene-Ramos M. (2016b). Modelling the Tox21 10 K chemical profiles for in vivo toxicity prediction and mechanism characterization. *Nat. Commun.* 7:10425. 10.1038/ncomms10425 26811972 PMC4777217

[B26] Huang S. Li X. Zhang Z. He Z. Wu F. Liu W. (2016c). Deep learning driven visual path prediction from a single image. *IEEE Trans. Image Process.* 25 5892–5904. 10.1109/TIP.2016.2613686 28114063

[B27] Hughes T. B. Dang N. L. Miller G. P. Swamidass S. J. (2016). Modeling reactivity to biological macromolecules with a deep multitask network. *ACS Cent. Sci.* 2 529–537. 10.1021/acscentsci.6b00162 27610414 PMC4999971

[B28] Hughes T. B. Miller G. P. Swamidass S. J. (2015). Modeling epoxidation of drug-like molecules with a deep machine learning network. *ACS Cent. Sci.* 1 168–180. 10.1021/acscentsci.5b00131 27162970 PMC4827534

[B29] Hughes T. B. Swamidass S. J. (2017). Deep learning to predict the formation of quinone species in drug metabolism. *Chem. Res. Toxicol.* 30 642–656. 10.1021/acs.chemrestox.6b00385 28099803 PMC5871348

[B30] Jeni L. A. Cohn J. F. De La Torre F. (2013). “Facing imbalanced data–recommendations for the use of performance metrics,” in *Proceedings of the 2013 Humaine Association Conference on Affective Computing and Intelligent Interaction* (Geneva: IEEE) 245–251. 10.1109/ACII.2013.47 PMC4285355 25574450

[B31] Kim S. Thiessen P. A. Cheng T. Yu B. Bolton E. E. (2018). An update on PUG-REST: RESTful interface for programmatic access to PubChem. *Nucleic Acids Res.* 46 W563–W570. 10.1093/nar/gky294 29718389 PMC6030920

[B32] Koutsoukas A. Monaghan K. J. Li X. Huan J. (2017). Deep-learning: investigating deep neural networks hyper-parameters and comparison of performance to shallow methods for modeling bioactivity data. *J. Cheminform.* 9:42. 10.1186/s13321-017-0226-y 29086090 PMC5489441

[B33] LeCun Y. Bengio Y. Hinton G. (2015). Deep learning. *Nature* 521 436–444. 10.1038/nature14539 26017442

[B34] Lenselink E. B. ten Dijke N. Bongers B. Papadatos G. van Vlijmen H. W. T. Kowalczyk W. (2017). Beyond the hype: deep neural networks outperform established methods using a ChEMBL bioactivity benchmark set. *J. Cheminform.* 9:45. 10.1186/s13321-017-0232-0 29086168 PMC5555960

[B35] Li Y. Idakwo G. Thangapandian S. Chen M. Hong H. Zhang C. (2018). Target-specific toxicity knowledgebase (TsTKb): a novel toolkit for in silico predictive toxicology. *J. Environ. Sci. Heal. Part C* 36 1–18. 10.1080/10590501.2018.1537148 30426823

[B36] Liu R. Madore M. Glover K. P. Feasel M. G. Wallqvist A. (2018). Assessing deep and shallow learning methods for quantitative prediction of acute chemical toxicity. *Toxicol. Sci.* 164 512–526. 10.1093/toxsci/kfy111 29722883

[B37] Mayr A. Klambauer G. Unterthiner T. Hochreiter S. (2016). DeepTox: toxicity prediction using deep learning. *Front. Environ. Sci.* 3:80 10.3389/fenvs.2015.00080

[B38] Mayr A. Klambauer G. Unterthiner T. Steijaert M. Wegner J. K. Ceulemans H. (2018). Large-scale comparison of machine learning methods for drug target prediction on ChEMBL. *Chem. Sci.* 9 5441–5451. 10.1039/c8sc00148k 30155234 PMC6011237

[B39] Min S. Lee B. Yoon S. (2016). Deep learning in bioinformatics. *Brief. Bioinform* 18 851–869. 10.1093/bib/bbw068 27473064

[B40] National Research Council (ed.) (2007). *Toxicity Testing in the 21st Century: A Vision and A Strategy.* Washington, DC: National Academies Press.

[B41] Névéol A. Zweigenbaum P. Section Editors for the Imia Yearbook Section on Clinical Natural Language Processing (2018). Expanding the diversity of texts and applications: findings from the section on clinical natural language processing of the international medical informatics association yearbook. *Yearb. Med. Inform.* 27 193–198. 10.1055/s-0038-1667080 30157523 PMC6115241

[B42] O’Boyle N. M. Banck M. James C. A. Morley C. Vandermeersch T. Hutchison G. R. (2011). Open babel: an open chemical toolbox. *J. Cheminform.* 3:33. 10.1186/1758-2946-3-33 21982300 PMC3198950

[B43] Organization for Economic Co-operation and Development [OECD] (2013). *Guidance Document on Developing and Assessing Adverse Outcome Pathways.* Paris: OECD environment, health and safety publications.

[B44] Pedregosa F. Varoquaux G. Gramfort A. Michel V. Thirion B. Grisel O. (2011). Scikit-learn: machine learning in python. *J. Mach. Learn. Res.* 12 2825–2830.

[B45] Plewczynski D. Spieser S. A. H. Koch U. (2006). Assessing different classification methods for virtual screening. *J. Chem. Inf. Model.* 46 1098–1106. 10.1021/ci050519k 16711730

[B46] Powers D. M. W. (2011). Evaluation: from precision, recall and F-Measure to roc, informedness, markedness & correlation. *J. Mach. Learn. Technol.* 2 37–63.

[B47] Saito T. Rehmsmeier M. Hood L. Franco O. Pereira R. Wang K. (2015). The precision-recall plot is more informative than the ROC plot when evaluating binary classifiers on imbalanced datasets. *PLoS One* 10:e0118432. 10.1371/journal.pone.0118432 25738806 PMC4349800

[B48] Shao C.-Y. Chen S.-Z. Su B.-H. Tseng Y. J. Esposito E. X. Hopfinger A. J. (2013). Dependence of QSAR models on the selection of trial descriptor sets: a demonstration using nanotoxicity endpoints of decorated nanotubes. *J. Chem. Inf. Model.* 53 142–158. 10.1021/ci3005308 23252880

[B49] Shen D. Wu G. Suk H.-I. (2017). Deep learning in medical image analysis. *Annu. Rev. Biomed. Eng.* 19 221–248. 10.1146/annurev-bioeng-071516-044442 28301734 PMC5479722

[B50] Silver D. Huang A. Maddison C. J. Guez A. Sifre L. van den Driessche G. (2016). Mastering the game of go with deep neural networks and tree search. *Nature* 529 484–489. 10.1038/nature16961 26819042

[B51] Snoek J. Larochelle H. Adams R. P. (2012). Practical bayesian optimization of machine learning algorithms. *Adv. Neural Inf. Process. Syst.* 25 2960–2968.

[B52] Stokes W. S. (2015). Animals and the 3Rs in toxicology research and testing: the way forward. *Hum. Exp. Toxicol.* 34 1297–1303. 10.1177/0960327115598410 26614819

[B53] Tan M. E. Li J. Xu H. E. Melcher K. Yong E. (2015). Androgen receptor: structure, role in prostate cancer and drug discovery. *Acta Pharmacol. Sin.* 36 3–23. 10.1038/aps.2014.18 24909511 PMC4571323

[B54] Thangapandian S. Shalini J. Sugunadevi S. Woo L. K. (2010). Docking-enabled pharmacophore model for histone deacetylase 8 inhibitors and its application in anti-cancer drug discovery. *J. Mol. Graph. Model.* 29 382–395. 10.1016/j.jmgm.2010.07.007 20870437

[B55] Tirado-Rives J. Jorgensen W. L. (2006). Contribution of conformer focusing to the uncertainty in predicting free energies for protein-ligand binding. *J. Med. Chem.* 49 5880–5884. 10.1021/jm060763i 17004703

[B56] Trott O. Olson A. J. (2010). AutoDock Vina: improving the speed and accuracy of docking with a new scoring function, efficientoptimization, and multithreading. *J. Comput. Chem.* 31 455–461. 10.1002/jcc 19499576 PMC3041641

[B57] Wu K. Wei G.-W. (2018). Quantitative toxicity prediction using topology based multitask deep neural networks. *J. Chem. Inf. Model.* 58 520–531. 10.1021/acs.jcim.7b00558 29314829

[B58] Wu Y. Wang G. (2018). Machine learning based toxicity prediction: from chemical structural description to transcriptome analysis. *Int. J. Mol. Sci.* 19:2358. 10.3390/ijms19082358 30103448 PMC6121588

[B59] Xu Y. Dai Z. Chen F. Gao S. Pei J. Lai L. (2015). Deep learning for drug-induced liver injury. *J. Chem. Inf. Model.* 55 2085–2093. 10.1021/acs.jcim.5b00238 26437739

[B60] Xu Y. Pei J. Lai L. (2017). Deep learning based regression and multiclass models for acute oral toxicity prediction with automatic chemical feature extraction. *J. Chem. Inf. Model.* 57 2672–2685. 10.1021/acs.jcim.7b00244 29019671

[B61] Yap C. W. (2011). PaDEL-descriptor: an open source software to calculate molecular descriptors and fingerprints. *J. Comput. Chem.* 32 1466–1474. 10.1002/jcc.21707 21425294

