# Supplementary material for: Deep Learning-Based Structure-Activity Relationship Modeling for Multi-Category Toxicity Classification: A Case Study of 10K Tox21 Chemicals With High-Throughput Cell-Based Androgen Receptor Bioassay Data
Source: Front Physiol. 2019 Aug 13;10:1044. doi: 10.3389/fphys.2019.01044 (PMC6700714; doi:10.3389/fphys.2019.01044)
Supplement: Supplementary file 1 [file Table_1.DOCX]

Supplementary Material

**Deep Learning-Based Structure-Activity Relationship Modeling for Multi-Category Toxicity Classification: A Case Study of 10K Tox21 Chemicals with High-Throughput Cell-Based Androgen Receptor Bioassay Data**

**Gabriel Idakwo^1#^, Sundar Thangapandian^2#^, Joseph Luttrell IV^1^, Zhaoxian Zhou^1^, Chaoyang Zhang^1*^, Ping Gong ^2*^**

^1^ School of Computing Sciences and Computer Engineering, University of Southern Mississippi, Hattiesburg, MS, USA.

^2^ Environmental Laboratory, U.S. Army Engineer Research and Development Center, Vicksburg, MS, USA.

^#^ These authors contributed equally to this work.

*** Correspondence:**

Dr. Ping Gong, [Ping.Gong@usace.army.mil](mailto:Ping.Gong@usace.army.mil)

Dr. Chaoyang Zhang, [Chaoyang.Zhang@usm.edu](mailto:Chaoyang.Zhang@usm.edu)


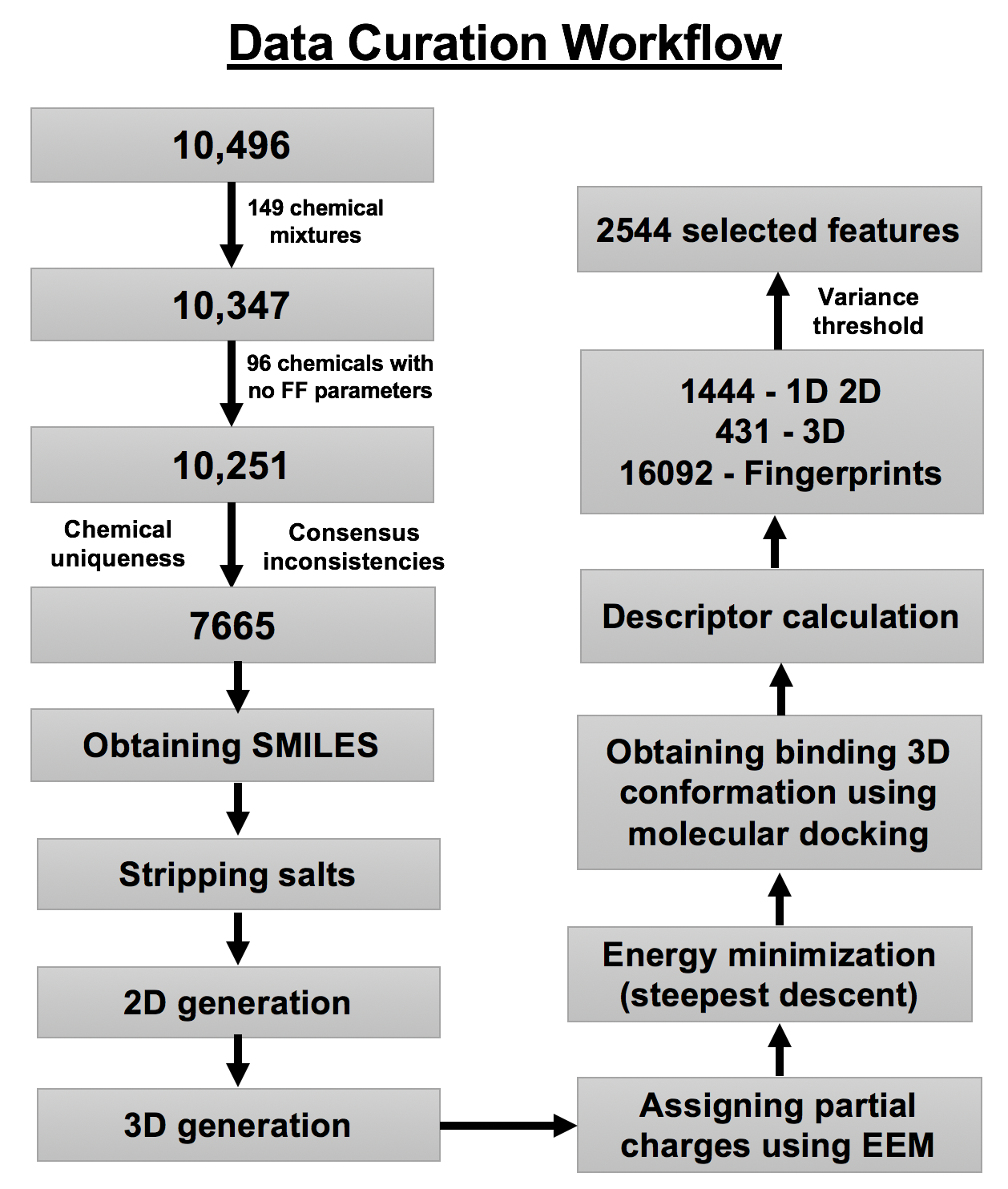


**Figure S1.** Data curation and preprocessing workflow.

FF = Force Field; EEM = Electronegativity Equalization Method.

Figure S2: Algorithm Spot check


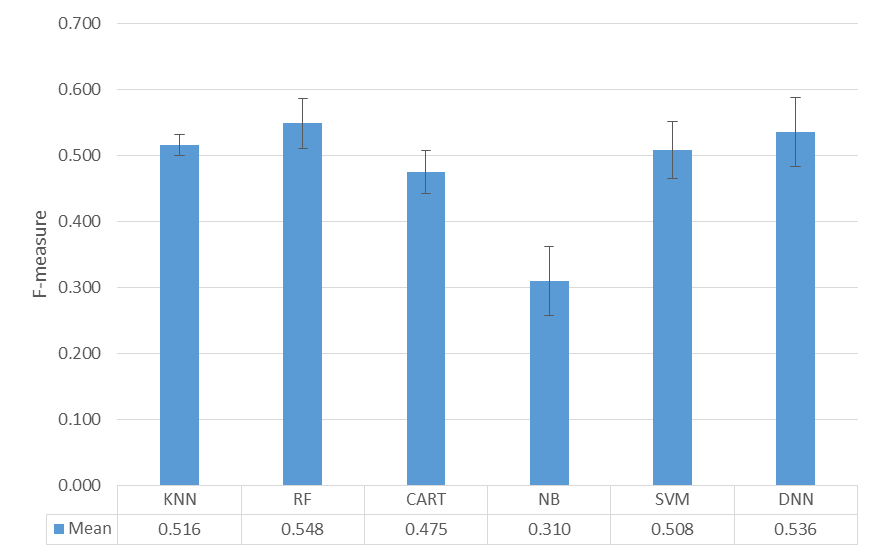


**Figure S2.** Preliminary comparative study results of the F-measure (mean ± standard deviation, n = 5) obtained from 5-fold cross-validation runs for 6 different machine learning algorithms that ran under their respective default settings as implemented in Scikit-Learn. See Figure 1 for the workflow. The same preprocessed data were used in the preliminary study as in subsequent definitive optimization studies on RF and DNN. KNN = k-nearest neighbor, RF = random forest, CART = classification and regression trees, NB = Naïve Bayes, SVM = support vector machine, and DNN = deep neural networks.

**
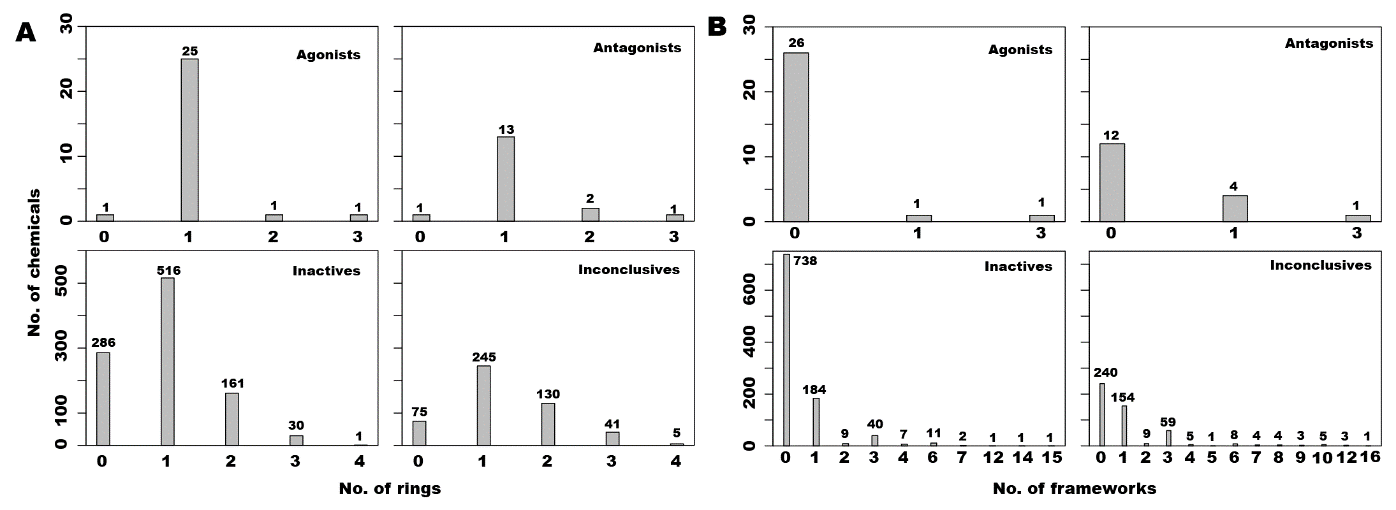
**

**Figure S3.** Number of rings (A) and frameworks (B) present in the four classes of Fold-1 chemicals.

**
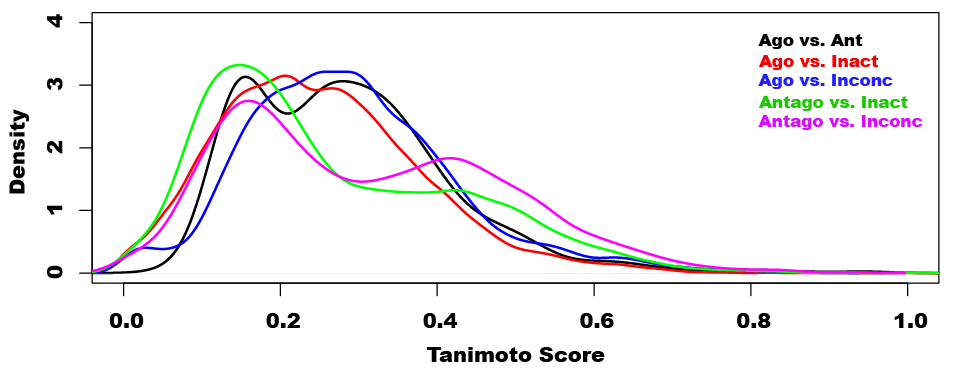
**

**Figure S4.** Kernel density plot displaying the distribution of inter-class chemical pairs over the full spectrum of Tanimoto Scores (TS) for the Fold-1 test chemicals. Density (y-axis) was calculated by (1) setting the TS bin width automatically using the R code, (2) placing chemical pairs in the bins, and (3) dividing the number of pairs in each bin by the total number of pairs and by the bin width. The plot shows that antagonists are more similar to inactive (green color) and inconclusive (magenta color) compounds than agonists (red and blue colors, respectively) are, if setting the TS threshold to 0.5.

**Table S1.** Parameter optimization for the random forest (RF) algorithm implemented in Scikit-Learn. Shown below are the initial distribution of five parameters considered for optimization and the final optimized values for these parameters that produced the best results.

| Parameters | Initial distribution | Optimized |
| --- | --- | --- |
| Max_depth | 2,3, None | None |
| Criterion | "gini", "entropy" | gini |
| Min_samples_leaf | 0.5, 1, 5, 10, 20,25 | 10 |
| N_estimators | 50, 100, 200, 300,400 | 200 |
| Max_features | "auto", "log2", None, 0.8, 0.5, 0.2,0.1 | 0.2 |
|  |  |  |

**Table S2.** Detailed performance report for DNN and RF using Fold 1 as the external test dataset.

**Table S3.** Detailed performance report for DNN and RF using Fold 2 as the external test dataset.

**Table S4.** Detailed performance report for DNN and RF using Fold 3 as the external test dataset.

**Table S5.** Detailed performance report for DNN and RF using Fold 4 as the external test dataset.

**Table S6.** Detailed performance report for DNN and RF using Fold 5 as the external test dataset.

**Table S7.** Macro-averages of five evaluation metrics derived from five external test subsets and one metric from the training set using **deep neural networks**. See Tables S2 to S6 for the raw confusion matrices and original macro-averages of metrics. AUROC = the area under ROC (receiver operating characteristics) curve; AURPC = the area under precision-recall curve

|  | Precision | Recall | F-measure | AUROC | AUPRC | Training (F-measure) |
| --- | --- | --- | --- | --- | --- | --- |
| Fold 1 | 0.855 | 0.846 | 0.847 | 0.969 | 0.890 | 0.91 |
| Fold 2 | 0.812 | 0.818 | 0.813 | 0.963 | 0.880 | 0.91 |
| Fold 3 | 0.860 | 0.773 | 0.808 | 0.957 | 0.843 | 0.91 |
| Fold 4 | 0.910 | 0.802 | 0.846 | 0.954 | 0.882 | 0.91 |
| Fold 5 | 0.894 | 0.822 | 0.848 | 0.955 | 0.883 | 0.90 |
| **Average** | **0.866** | **0.812** | **0.832** | **0.960** | **0.876** | **0.908** |
| Std. Dev | 0.034 | 0.024 | 0.018 | 0.006 | 0.017 | 0.004 |
| CV (%) | 3.9 | 3.0 | 2.2 | 0.6 | 1.9 | 0.4 |

**Table S8.** Macro-averages of five evaluation metrics derived from five external test subsets and one metric from the training set using **random forest**. See Tables S2 to S6 for the raw confusion matrices and original macro-averages of metrics. AUROC = the area under ROC (receiver operating characteristics) curve; AURPC = the area under precision-recall curve)

|  | Precision | Recall | F-measure | AUROC | AUPRC | Training (F-measure) |
| --- | --- | --- | --- | --- | --- | --- |
| Fold 1 | 0.711 | 0.556 | 0.598 | 0.846 | 0.643 | 0.751 |
| Fold 2 | 0.534 | 0.542 | 0.536 | 0.866 | 0.667 | 0.746 |
| Fold 3 | 0.571 | 0.512 | 0.536 | 0.857 | 0.599 | 0.750 |
| Fold 4 | 0.580 | 0.535 | 0.552 | 0.827 | 0.594 | 0.747 |
| Fold 5 | 0.820 | 0.591 | 0.599 | 0.841 | 0.644 | 0.752 |
| **Average** | **0.643** | **0.547** | **0.564** | **0.847** | **0.629** | **0.749** |
| Std. Dev | 0.107 | 0.026 | 0.029 | 0.013 | 0.028 | 0.002 |
| CV (%) | 16.6 | 4.8 | 5.1 | 1.5 | 4.5 | 0.3 |
